# FOXM1 confers resistance to gefitinib in lung adenocarcinoma via a MET/AKT-dependent positive feedback loop

**DOI:** 10.18632/oncotarget.11043

**Published:** 2016-08-03

**Authors:** Yu Wang, Weiwei Zhang, Li Wen, Huiling Yang, Mingling Wen, Yuyu Yun, Lisheng Zhao, Xiaofei Zhu, Li Tian, Erping Luo, Yu Li, Wenchao Liu, Ning Wen

**Affiliations:** ^1^ Department of Oncology, State Key Discipline of Cell Biology, Xijing Hospital, The Fourth Military Medical University, Xi'an, Shaanxi, China; ^2^ Institute of Stomatology, Chinese PLA General Hospital, Beijing, China; ^3^ Department of Biomedical Engineering, The Fourth Military Medical University, Xi'an, Shaanxi, China; ^4^ Department of Pharmacy, Affiliated Hospital of Academy of Military Medical Sciences, Beijing, China; ^5^ State Key Laboratory of Cancer Biology, Cell Engineering Research Center and Department of Cell Biology, The Fourth Military Medical University, Xi'an, Shaanxi, China; ^6^ Department of Neurology, Kunming General Hospital, Chinese People's Liberation Army, Kunming, Yunnan, China; ^7^ Department of Anesthesiology, Xijing Hospital, The Fourth Military Medical University, Xi'an, Shaanxi, China

**Keywords:** FOXM1, MET, AKT, lung adenocarcinoma, gefitinib

## Abstract

Gefitinib resistance remains a major problem in the treatment of lung adenocarcinoma. However, the molecular mechanisms of gefitinib resistance are not fully understood. In this study, we characterized the critical role of transcription factor Forkhead box protein M1 (FOXM1) in gefitinib resistance of lung adenocarcinoma cells. *In vitro* drug sensitivity assays demonstrated that FOXM1 inhibition sensitized PC9/GR and HCC827/GR cells to gefitinib, whereas FOXM1 overexpression enhanced PC9 and HCC827 cell resistance to gefitinib. Increased FOXM1 resulted in the upregulation of hepatocyte growth factor receptor (MET), which led to activation of the protein kinase B (AKT) pathway, whereas knockdown of FOXM1 did the opposite. FOXM1 bound directly to the MET promoter regions and regulated the promoter activities and the expression of MET at the transcriptional level. Moreover, MET/AKT pathway upregulated the expression of FOXM1 in lung adenocarcinoma cells. Inhibition of pAKT by LY294002 or inhibition of pMET by PHA-665752 significantly inhibited the expression of FOXM1 in lung adenocarcinoma cells. Importantly, we further demonstrated that the expression levels of FOXM1, pAKT and MET were significantly increased in lung adenocarcinoma tissues relative to normal lung tissues, and these three biomarkers were concomitantly overexpressed in lung adenocarcinoma tissues. Taken together, our results indicate that FOXM1 promotes acquired resistance to gefitinib of lung adenocarcinoma cells, and FOXM1 crosstalks with MET/AKT signaling to form a positive feedback loop to promote lung adenocarcinoma development.

## INTRODUCTION

According to new statistics offered by the American Cancer Society, lung cancer is the first most common cause of cancer-related death and the most lethal cancer in the United States [1]. In 2015, it is estimated that new cases and deaths from lung cancer in the United States was 221,200 and 158,040, respectively [1]. Adenocarcinoma is the most common type of lung cancer accounting for 40% of all cases with its incidence increasing [2, 3]. Gefitinib is a specific and effective epidermal growth factor receptor tyrosine kinase inhibitors (EGFR-TKIs), and it has been studied and used to treat lung adenocarcinoma patients with activating EGFR mutations, such as exon 19 deletion and exon 21 L858R mutation [4, 5]. However, despite initial dramatic response in patients harboring such EGFR mutations, most patients will eventually develop acquired resistance to EGFR-TKIs [6–8]. Several mechanisms underlying acquired resistance to EGFR-TKIs have been identified, including secondary EGFR T790M mutation, MET amplification and HER2 amplification [9–11]. Elucidation of novel mechanisms underlying the acquired resistance to EGFR-TKIs is important for developing personalized therapeutics to treat patients with lung adenocarcinoma.

As a typical proliferation-associated transcription factor, FOXM1 mainly exerts its function in tumorigenesis through transcriptional regulation of its target genes to initiate various cellular responses, including cell growth, proliferation, differentiation, longevity and transformation [12–14]. It belongs to a large family of evolutionary conserved transcription factors that were characterized by a conserved DNA binding domain called Forkhead or winged-helix domain [14–16]. FOXM1 is frequently overexpressed in many human cancers, and its expression is associated with poor cancer outcomes [14, 17–23]. Previous studies have shown that FOXM1 is broadly involved in drug resistance [23–28]. Furthermore, several studies indicates that FoxM1 plays an important role in the resistance of cancer cells to gefitinib [29–32]. However, the molecular mechanisms underlying cancer cells resistance to gefitinib are still not fully understood.

Approximately 20–25% of EGFR-TKI resistance arises due to the amplification of MET, a gene that encodes a receptor tyrosine kinase for hepatocyte growth factor [33, 34]. The MET signaling pathway has been implicated in the development and progression of various cancers including lung cancer, but the mechanisms regulating its expression are not fully understood [35]. The MET amplification results in continuous activation of the phosphoinosmde-3-kinase (PI3K)/AKT signaling pathway, which is the major downstream signaling routes of EGFR [35]. Like the MET signaling pathway, which is also aberrantly activated in lung cancer, the PI3K/AKT pathway is strongly activated and plays a very important role in lung cancer development as well as EGFR-TKI resistance [36]. Moreover, the function of FOXM1 was reported to be mediated by PI3K/AKT signaling pathway [21, 37–39]. These findings suggest that MET/AKT/FOXM1 signaling pathway may play an important role in the resistance of lung adenocarcinoma cells to gefitinib.

In the study, we investigate the role of FOXM1 in the resistance of lung adenocarcinoma cells to gefitinib, and define a novel regulatory pathway directly linking the MET/AKT signaling axis to FOXM1. We found that FOXM1 bound directly to the promoter regions of MET and regulated expression of MET at the transcriptional level. We also found that FOXM1 was regulated by MET through PI3K/AKT signaling pathway and that the feedback regulation loop between FOXM1 and MET/AKT signaling pathway could play a pivotal role in gefitinib resistance of lung adenocarcinoma cells. Moreover, we demonstrated that expression levels of FOXM1, MET and pAKT were significantly higher in lung adenocarcinoma tissues than in normal lung tissues, and these three biomarkers were concomitantly overexpressed in lung adenocarcinoma tissues. Thus, our findings elucidated a novel FOXM1/MET/AKT regulatory feedback loop and identified that FOXM1 might be a target for overcoming gefitinib resistance in lung adenocarcinoma.

## RESULTS

### Characteristics of gefitinib-resistant lung adenocarcinoma cells

**Figure 1:**
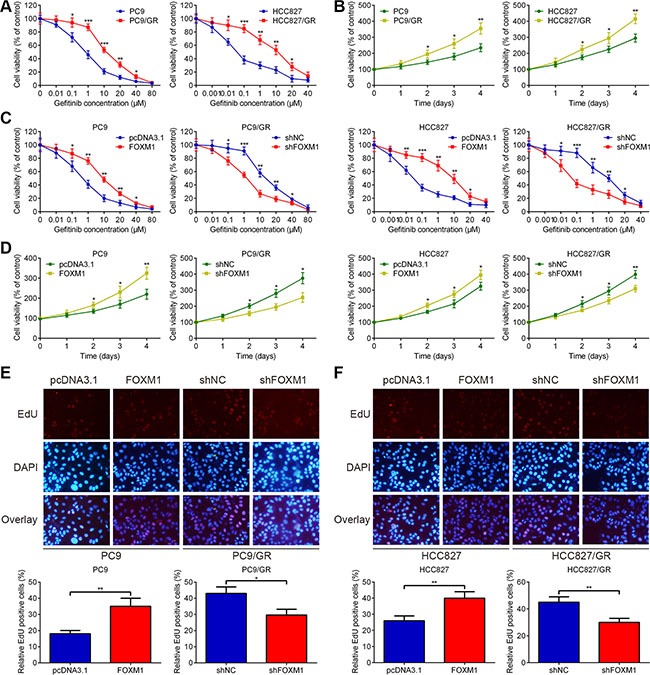
FOXM1 counteracts gefitinib-induced cell death of lung adenocarcinoma cells (**A**) PC9/GR and HCC827/GR were more resistant to gefitinib than their parental cells, respectively. (**B**) Cell proliferation rates were detected using MTT assay for four days, following treatment with gefitinib. (**C**) PC9, PC9/GR, HCC827 and HCC827/GR cells were transfected with negative control shRNA (shNC), pcDNA3.1 control vector (pcDNA3.1), pcDNA3.1-FOXM1 (FOXM1) or FOXM1 shRNA (shFOXM1) for 48 hrs, then treated with various concentrations of gefitinib for 72 hrs, and cell viability was analyzed using MTT assay. (**D**) Time-dependent effects of FOXM1 on the proliferation of PC9, PC9/GR, HCC827 and HCC827/GR cells were confirmed using MTT assay, following treatment with gefitinib. (**E** and **F**) EdU staining for evaluation of the influences of FOXM1 on the proliferation of lung adenocarcinoma cells. Cells were exposed to gefitinib for 72 hrs and subjected to EdU incorporation assays. The new generation cells were detected via EdU (red). DAPI stained nuclei in blue. Merged view of EdU (red) and DAPI (blue) showing the overlap. Each bar represents the mean ± SD. *P* values were calculated using Student's *t-test* (*P < 0.05, ** *P* < 0.01, *** *P* < 0.001).

Both PC9 and HCC827 cells had exon 19 deletion (E746-A750 deletion) in EGFR and showed strong sensitivity to gefitinib. To develop an *in vitro* model of acquired gefitinib resistance, we continuously exposed PC9 and HCC827 cells to gefitinib. After approximately 6 months of exposure, gefitinib-resistant PC9 (PC9/GR) and gefitinib-resistant HCC827 (HCC827/GR) cells were established. When we analyzed the EGFR mutational status in the exon 18 to 21 by performing sequencing, there was no difference between the PC9 and PC9/GR cells, and between the HCC827 and HCC827/GR cells. Compared with parental PC9 and HCC827 cells, PC9/GR and HCC827/GR cells are larger in size and have irregular distributions before cell fusion. Acquired resistance to gefitinib was confirmed by MTT assays for PC9/GR and HCC827/GR cells. As shown in Figure 1A and 1B, PC9/GR and HCC827/GR cells were significantly resistant to gefitinib compared to parental PC9 and HCC827 cells in a dose or time-dependent manner, respectively. The IC50 value of gefitinib in PC9 cells was 0.74 ± 0.11 μM, compared to 13.66 ± 0.62 μM in PC9/GR cells. The IC50 value of gefitinib in HCC827 cells was 0.04 ± 0.01 μM, compared to 10.06 ± 0.43 μM in HCC827/GR cells. Predominant accumulation in S phase was observed in PC9/GR and HCC827/GR cells compared with PC9 and HCC827 cells, respectively. No significant deviation in apoptosis was observed.

### FOXM1 mediates gefitinib resistance in lung adenocarcinoma cells

To test the significance of FOXM1 interference in lung adenocarcinoma cells, we transfected pcDNA3.1-FOXM1 plasmid into PC9 and HCC827 cells, and transfected FOXM1 shRNA into PC9/GR and HCC827/GR cells. Western blot and qRT-PCR assays were performed to confirm the transfection efficiency. As shown in Figure 1C and 1D, FOXM1 overexpression promoted PC9 and HCC827 cell resistance to gefitinib treatment, whereas knockdown of FOXM1 increased gefitinib sensitivity of PC9/GR and HCC827/GR cells. In addition, we determined the effect of FOXM1 on DNA synthesis and cell proliferation using an EdU assay. Compared to the pcDNA3.1 group, the number of EdU-positive cells significantly increased upon FOXM1 overexpression, suggesting that FOXM1 overexpression increased the DNA synthesis upon gefitinib treatment (Figure 1E and 1F). Simultaneously, compared to the shNC group, the number of EdU-positive cells significantly decreased upon FOXM1 knockdown, suggesting that FOXM1 knockdown inhibited the DNA synthesis upon gefitinib treatment (Figure 1E and 1F). Taken together, these results strongly suggested that FOXM1 was involved in mediating the response to gefitinib in lung adenocarcinoma cell lines.

### FOXM1 reduces G1 arrest and apoptosis of lung adenocarcinoma cells following gefitinib exposure

**Figure 2:**
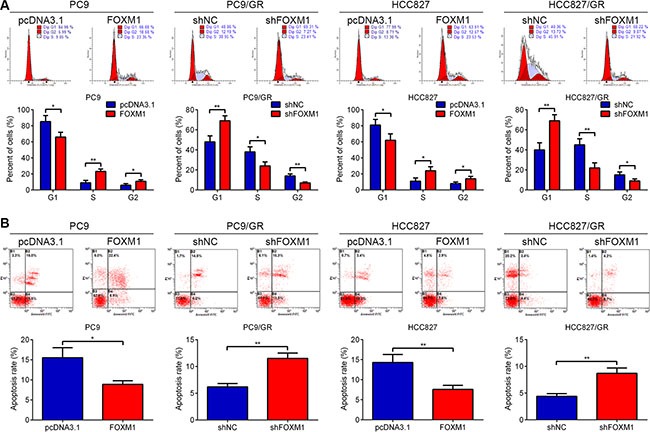
FOXM1 promoted cell proliferation and inhibited apoptosis in lung adenocarcinoma cells upon gefitinib treatment PC9, PC9/GR, HCC827 and HCC827/GR cells transfected with pcDNA3.1-FOXM1 (FOXM1) or FOXM1 shRNA (shFOXM1) were treated with gefitinib for 72 hrs. Flow cytometric analysis of cell cycle (**A**) and apoptosis (**B**) indicated that FOXM1 down-regulation significantly induced cell apoptosis and cell cycle arrest in G1 phase, vice versa. Each Column was mean of three independent experiments. Each bar represents mean ± SD. *P < 0.05, **P < 0.01 by Student's *t-test*.

We examined gefitinib-induced cell cycle arrest and apoptosis in PC9, HCC827, PC9/GR and HCC827/GR cells following pcDNA3.1-FOXM1 or FOXM1 shRNA transfection. As shown in Figure 2A, FOXM1 overexpression resulted in a decreased percentage of PC9 and HCC827 cells in G1 phase, whereas down-regulation of FOXM1 triggered PC9/GR and HCC827/GR cell cycle arrest in G1 phase. In addition, a significant decrease in apoptosis was observed in PC9 and HCC827 cells transfected with FOXM1 after gefitinib treatment compared with control transfected cells, whereas a marked increase in apoptosis was found in PC9/GR and HCC827/GR cells transfected with FOXM1 shRNA after gefitinib treatment compared with control transfected cells (Figure 2B). These data clearly indicated that FOXM1 overexpression in lung adenocarcinoma cells attenuated cell apoptosis and G1 arrest effects of gefitinib.

### FOXM1 activates the AKT pathway through MET in lung adenocarcinoma cells

**Figure 3:**
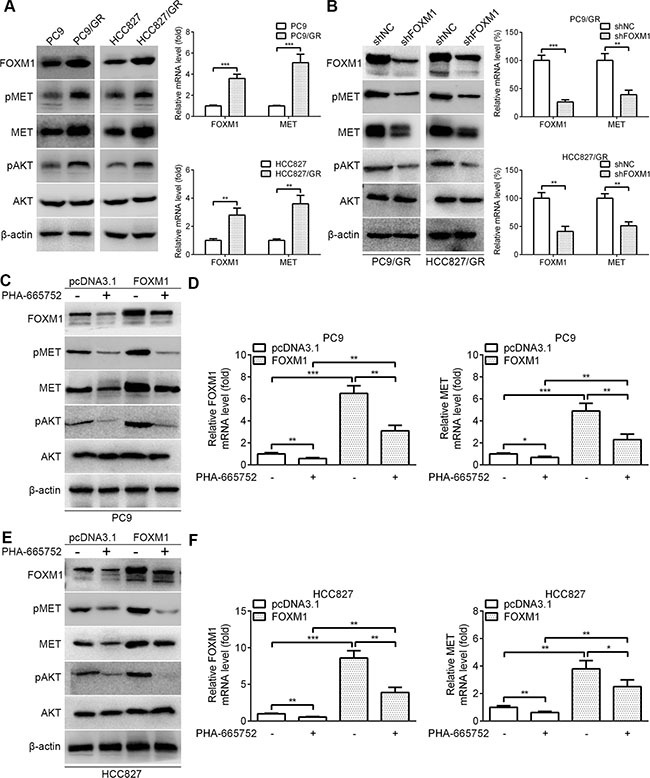
FOXM1 activates pAKT signaling via MET in lung adenocarcinoma cells (**A**) The basal protein expressions of FOXM1, pMET, MET, pAKT and AKT in PC9, HCC827, PC9/GR and HCC827/GR cells were determined by western blot analysis. The basal mRNA expressions of FOXM1 and MET in PC9, HCC827, PC9/GR and HCC827/GR cells were determined by quantitative real-time PCR analysis. (**B**) PC9/GR and HCC827/GR cells were transfected with FOXM1 shRNA or shNC, the protein levels of FOXM1, pMET, MET, pAKT and AKT were analyzed by western blot analysis, and the mRNA levels of FOXM1 and MET were analyzed by quantitative real-time PCR analysis. (**C**–**F**) PC9-FOXM1 and HCC827-FOXM1 cells were treated with PHA-665752 for 6 hours, the protein levels of FOXM1, pMET, MET, pAKT and AKT were analyzed by western blot analysis, and the mRNA levels of FOXM1 and MET were analyzed by quantitative real-time PCR analysis. Data are presented as the mean ± SD. *P < 0.05, **P < 0.01, ***P < 0.001, Student's *t-test*.

We examined the expression levels of FOXM1 and related molecules in lung adenocarcinoma cells by western blot and quantitative real-time PCR. Notably, the expression levels of FOXM1, pMET, MET and pAKT were increased in PC9/GR and HCC827/GR cells relative to those in the parental cells, respectively (Figure 3A). We next explored the regulatory relationship between FOXM1 and the MET/AKT signaling pathway. To determine whether FOXM1 is a key mediator of the MET/AKT signaling pathway, negative control shRNA (shNC) and FOXM1 shRNA (shFOXM1) were transfected into PC9/GR and HCC827/GR cells. As shown in Figure 3B, western blot assays showed that the protein levels of FOXM1, pMET, MET and pAKT were significantly decreased by FOXM1 knockdown in PC9/GR and HCC827/GR cells. In line with our above results, quantitative real-time PCR analyses showed that FOXM1 and MET mRNA levels were significantly decreased by FOXM1 knockdown in PC9/GR and HCC827/GR cells (Figure 3B). To investigate whether the activation of the AKT pathway by FOXM1 depends on MET, we used PHA-665752 (a potent and selective inhibitor of MET) to block MET phosphorylation in PC9 and HCC827 cells. Our results showed that FOXM1 transfection significantly upregulated the expressions of FOXM1, pMET, MET and pAKT, but this effect was partially inhibited by PHA-665752 treatment (Figure 3C–3F). Collectively, these results indicate that FOXM1 activates the AKT pathway through MET in lung adenocarcinoma cells.

### FOXM1 is a transcriptional activator of MET

**Figure 4:**
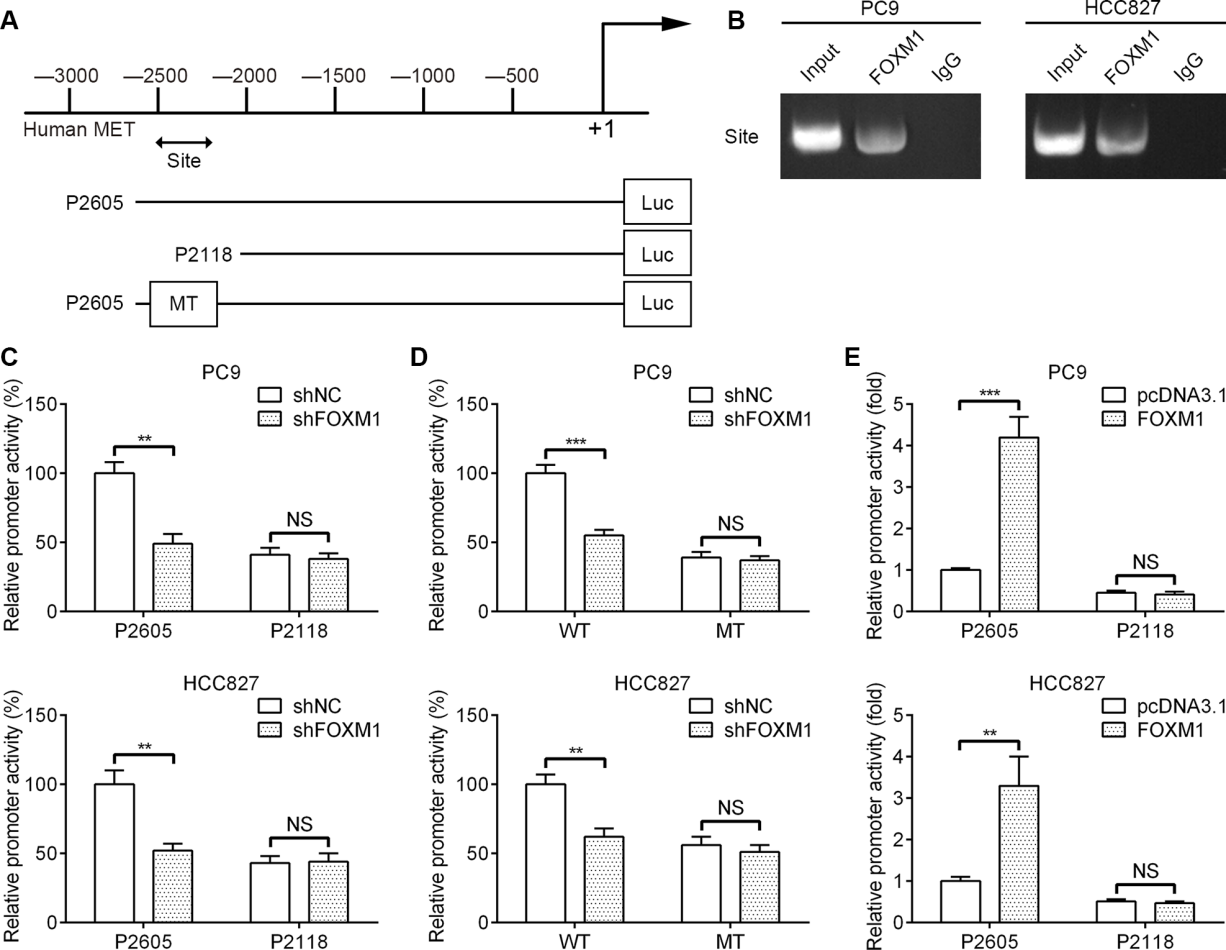
FOXM1 binds to human MET promoter and directly enhances its transcription (**A**) A putative FOXM1-binding site in the MET promoter and construction of reporter plasmids. (**B**) ChIP analysis of the MET promoter using antibodies against FOXM1 in PC9 and HCC827 cells. (**C**) The promoter activity of two truncated constructs was measured in PC9 and HCC827 cells when cotransfected with the control plasmid or FOXM1 shRNA plasmid. (**D**) The transcriptional activity of FOXM1 on MET-luc wide type (WT) or mutants (MT) was analyzed by luciferase reporter assay in PC9 and HCC827 cells. (**E**) The promoter activity of two truncated constructs was measured in PC9 and HCC827 cells when cotransfected with the control plasmid or pcDNA3.1-FOXM1 plasmid. Promoter activity was examined using a dual luciferase assay kit. The data represent three independent experiments, each bar represents mean ± SD. *P* values were calculated using Student *t-test* (**P < 0.01, ***P < 0.001, NS, nonsignificant).

To dissect the molecular mechanism of the effects of FOXM1 on MET expression, we analyzed the sequences of MET promoter for the potential FOXM1-binding elements. Intriguingly, we identified a putative FOXM1-binding element in the MET promoter region (Figure 4A). To explore whether FOXM1 directly regulates MET, we first performed ChIP assays in PC9 and HCC827 cells. The results suggested that MET chromatins were specifically immunoprecipitated with antibody against FOXM1, compared with the IgG control (Figure 4B). Moreover, a series of reporter gene constructs based on the potential binding sites were generated (Figure 4A). These reporter constructs were cotransfected into PC9 and HCC827 cells with FOXM1 shRNA, pcDNA3.1–FOXM1 or control vector. As shown in Figure 4C, knockdown of FOXM1 significantly decreased the MET promoter activity in the P2605 construct, and altered expression of FOXM1 did not change the promoter activity in the P2118 construct, which did not contain the potential FOXM1 binding site. We mutated the putative binding sites within the luciferase reporter constructs (Figure 4A). As shown in Figure 4D, knockdown of FOXM1 significantly reduced the activity of the WT (wild-type) pLuc-MET construct in PC9 and HCC827 cells, and altered expression of FOXM1 did not change the activity of the MT (mutant) pLuc-MET construct. Additionally, FOXM1 overexpression markedly increased the MET promoter activity in the P2605 construct, and altered expression of FOXM1 did not change the promoter activity in the P2118 construct (Figure 4E). Collectively, these results support that FOXM1 is an authentic and direct transcriptional activator for MET.

### MET regulates FOXM1 through the AKT pathway

**Figure 5:**
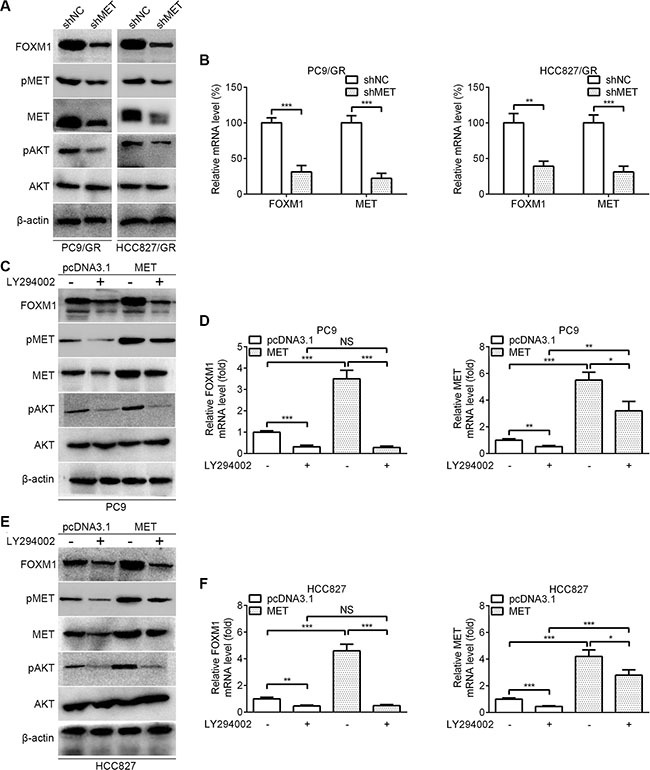
FOXM1 is activated by MET/AKT signaling pathway (**A**) Western blot analysis for FOXM1, pMET, MET, pAKT and AKT in PC9/GR and HCC827/GR that were transfected with MET shRNA. (**B**) Quantitative real-time PCR analysis for FOXM1 and MET in PC9/GR and HCC827/GR that were transfected with MET shRNA. (**C**–**F**) PC9-MET and HCC827-MET cells were treated with LY294002 for 6 hours, the protein levels of FOXM1, pMET, MET, pAKT and AKT were analyzed by western blot analysis, and the mRNA levels of FOXM1 and MET were analyzed by quantitative real-time PCR analysis. Each bar represents the mean ± SD. *P* values were calculated using Student's *t-test* (*P < 0.05, **P < 0.01, ***P < 0.001, NS, nonsignificant).

To further reveal the crosstalk between FOXM1 and the AKT pathway, we examined the effect of the MET/AKT signaling pathway on FOXM1. As shown in Figure 5A, western blot assays showed that the protein levels of FOXM1, pMET, MET and pAKT were significantly decreased by MET knockdown in PC9/GR and HCC827/GR cells. As shown in Figure 5B, quantitative real-time PCR analyses showed that FOXM1 and MET mRNA levels were significantly decreased by MET knockdown in PC9/GR and HCC827/GR cells. To dissect whether MET regulates FOXM1 through the AKT pathway, we examined FOXM1 expression after inhibition of PI3K/AKT pathway using LY294002. As shown in Figure 5C–5F, our results showed that MET transfection significantly upregulated the expressions of FOXM1, pMET, MET and pAKT, but this effect was partially inhibited by LY294002 treatment. These data combined with that FOXM1 activates the AKT pathway through MET demonstrate that there exists a positive feedback regulation between FOXM1 and the MET/AKT signaling pathway in lung adenocarcinoma cells.

### FOXM1 mediates gefitinib resistance of lung adenocarcinoma cells in a xenograft model

**Figure 6:**
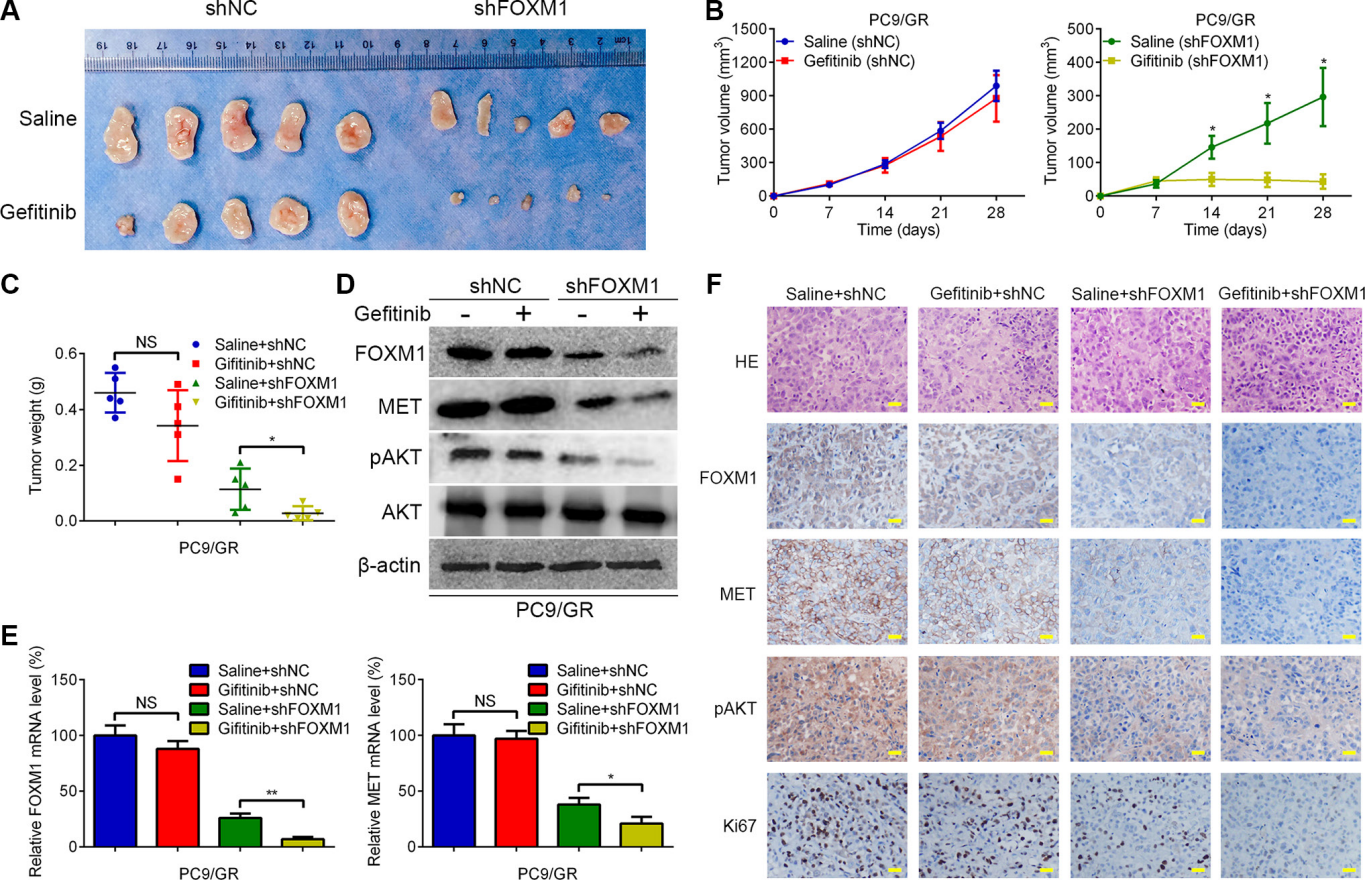
FOXM1 knockdown enhances gefitinib sensitivity *in vivo* (**A**) Representative pictures of xenograft tumors are shown. (**B**) The tumor volumes were measured at the indicated time points. (**C**) Tumor weight derived from FOXM1-shRNA knockdown or control-shRNA knockdown was measured at day 28. (**D**–**F**) The expression levels of FOXM1, MET and pAKT were analyzed by qRT–PCR, western blotting and immunohistochemistry. Data are represented as means ± SD of each group. *P < 0.05, **P < 0.01, NS, nonsignificant, Student's *t-test*.

To further confirm the role of FOXM1 in gefitinib resistance in an *in vivo* xenograft model, the stable shFOXM1 and shNC-transfected PC9/GR cells were injected subcutaneously into BALB/C nude mice. One week after inoculation, gefitinib was administered via oral gavage at a dose of 20 mg/kg every day. By 4 weeks, gefitinib monotherapy did not have significant effects on PC9/GR xenograft, in contrast to the saline group (Figure 6A–6C). In contrast, the combination of FOXM1 knockdown and gefitinib led to marked inhibition of the tumor growth compared with either FOXM1 knockdown or gefitinib monotherapy (Figure 6A–6C). Western blot and qRT-PCR analyses showed that compared with either FOXM1 knockdown or gefitinib monotherapy group, the expressions of FOXM1, MET and pAKT were significantly decreased in the FOXM1 knockdown plus gefitinib treatment group, which was further confirmed by immunohistochemical examination of xenograft tumor sections (Figure 6D–6F). Immunohistochemical analysis also showed that the cell proliferation marker Ki67 was significantly downregulated in the FOXM1 knockdown plus gefitinib treatment group. Collectively, these results suggest that knockdown of FOXM1 is a useful treatment to overcome gefitinib resistance *in vivo*.

### Correlation of FOXM1, pAKT and MET expression in lung adenocarcinoma patients

**Figure 7:**
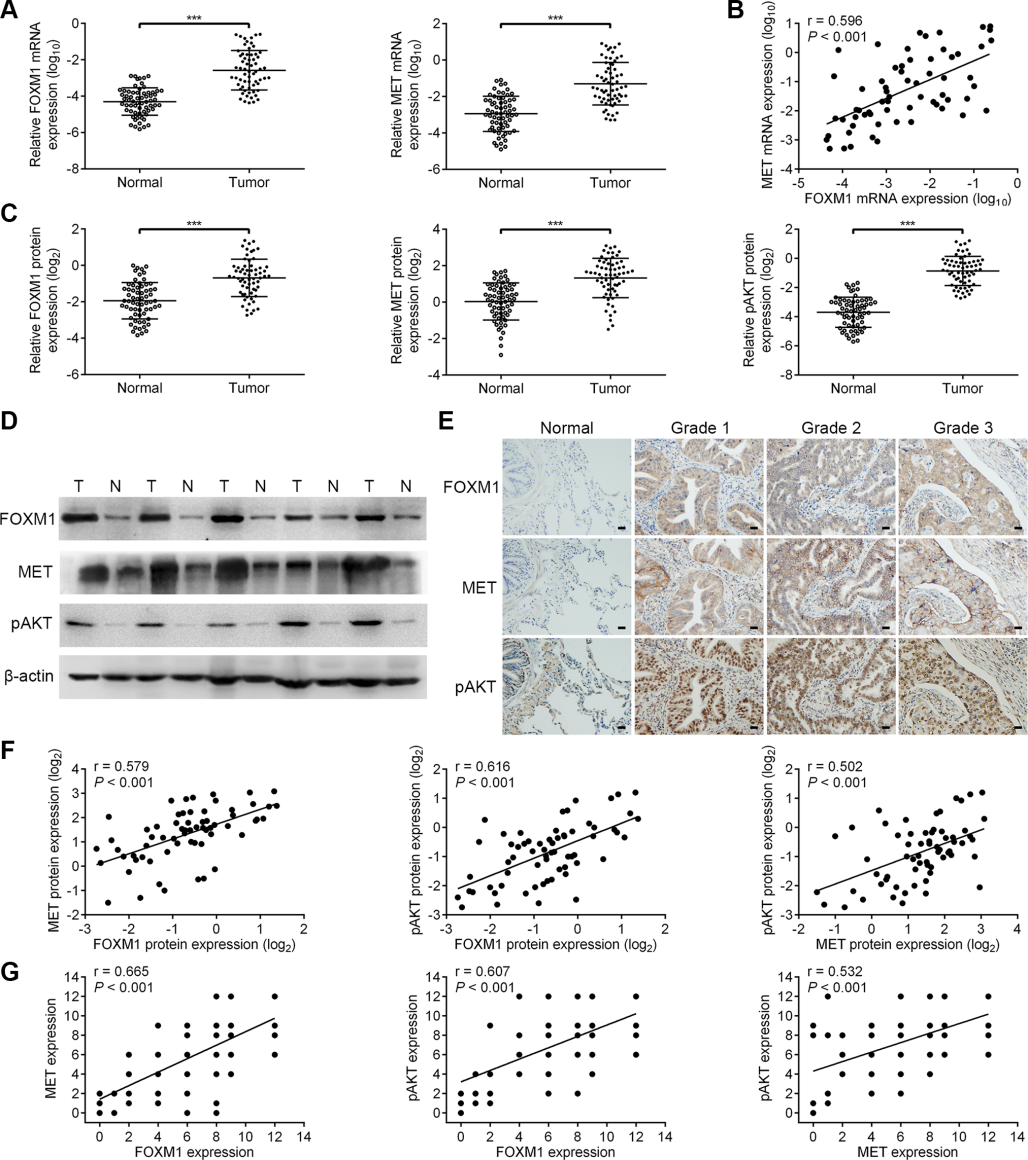
The coordinate expression of FOXM1, MET and pAKT in lung adenocarcinoma tissues (**A**) Relative mRNA levels of FOXM1 and MET were assessed by qRT-PCR analysis in 64 pairs of lung adenocarcinoma tissues and corresponding nontumorous tissues. (**B**) Scatter plot analysis of correlation between mRNA expression levels of FOXM1 and MET in 64 lung adenocarcinoma tissues. (**C** and **D**) Relative protein levels of FOXM1, MET and pAKT were assessed by western blot analysis in 64 pairs of lung adenocarcinoma tissues and corresponding nontumorous tissues. (**E**) Representative immunohistochemical staining images of FOXM1, MET and pAKT by using consecutive tissue sections from same lung adenocarcinoma patient (Scale bars, 100 μm). (**F**) Scatter plot analysis of correlation between protein expression levels of FOXM1, MET and pAKT in 64 lung adenocarcinoma tissues. (**G**) The relationship between the expression of FOXM1, MET and pAKT was analyzed based on IHC staining. Note that some of the dots on the graphs represent more than one specimen. Data are represented as means ± SD of each group. ***P < 0.001, Student's *t-test*.

To determine whether our findings have clinical relevance in human lung adenocarcinoma, we investigated the expression of FOXM1, MET and pAKT in human lung adenocarcinoma samples. We analyzed the significance of the FOXM1/MET/AKT axis in a total of 64 pairs of lung adenocarcinoma specimens and adjacent non-cancerous specimens. As shown in Figure 7A, the mRNA expressions of FOXM1 and MET were confirmed to be higher in lung adenocarcinoma specimens than in adjacent non-cancerous specimens. Importantly, we observed that tumors exhibiting high FOXM1 mRNA expression also expressed elevated mRNA levels of MET, indicating that a positive correlation between FOXM1 mRNA and MET mRNA levels (Figure 7B). Also, in the same set of lung adenocarcinoma specimens and adjacent non-cancerous specimens, we examined the expressions of FOXM1, MET and pAKT by western blot and immunohistochemical analyses (Figure 7C–7E). As shown in Figure 7C–7E, the protein expressions of FOXM1, MET and pAKT were confirmed to be higher in lung adenocarcinoma specimens than in adjacent non-cancerous specimens. Moreover, Spearman rank correlation analysis showed significant positive correlations between FOXM1 and MET protein levels, FOXM1 and pAKT protein levels, MET and pAKT protein levels (Figure 7F and 7G). These results support our finding that FOXM1, MET and pAKT are strictly coexpressed in lung adenocarcinoma specimens and our findings in model systems find a close parallel in clinical samples.

## DISCUSSION

Gefitinib resistance is a major obstacle in clinical practice leading to an overall poor outcome and reduced patient survival, yet the mechanisms underlying resistance to specific therapies have been largely unexplored. Establishment of drug-resistant sublines and comparative investigations with their parental cells are useful approaches that can be used to identify functional molecules involved in the mechanism of drug resistance. In the present study, two gefitinib-sensitive lung adenocarcinoma cell lines and their sub-lines with enhanced gefitinib resistance were studied to systemically address the role of FOXM1 in gefitinib resistance of lung adenocarcinoma cells. First, we found that FOXM1 was involved in mediating the response to gefitinib, and attenuated cell apoptosis and G1 arrest effects of gefitinib in lung adenocarcinoma cells. Second, the expression of total MET, pMET, pAKT, and FOXM1, but not total AKT expression, were significantly increased in gefitinib-resistant cells compared with sensitive cells. Third, FOXM1 bound directly to the promoter regions of MET and regulated expression of MET at the transcriptional level. Fourth, FOXM1 was a downstream target of AKT signalling, and there was a positive feedback regulation between FOXM1 and the MET/AKT signaling pathway in lung adenocarcinoma cells. Finally, we found that FOXM1, MET and pAKT were concomitantly overexpressed in lung adenocarcinoma specimens. These results clearly indicate that the regulatory feedback between FOXM1 and MET/AKT pathway may represent a critical mechanism of gefitinib resistance of lung adenocarcinoma cells.

FOXM1 is well-known for its critical role in cell cycle progression by regulating the transition from G1 to S phase and G2 to M phase progression, as well as to mitosis [14]. Besides its essential roles in cell cycle regulation, FOXM1 also emerged as an oncogenic transcription factor with a high expression and functional impact in many types of cancer cells [14, 17–23]. Furthermore, previous studies indicate that FOXM1 induces chemotherapy resistance in various cancer cells by protecting cancer cell proliferation and abrogating apoptosis [23–28]. Gefitinib, a selective and effective EGFR tyrosine kinase inhibitor, significantly inhibits EGFR autophosphorylation and subsequent downstream signaling pathway through AKT, and induces G1 cell cycle arrest [40]. Previous studies have confirmed that FOXM1 was a downstream cellular target of EGFR signaling and had an important role in cancer development [41, 42]. Therefore, we hypothesized that FOXM1 might be involved in gefitinib resistance of lung adenocarcinoma cells. Here we showed that FOXM1 overexpression promoted PC9 and HCC827 cell resistance to gefitinib treatment, whereas FOXM1 knockdown increased gefitinib sensitivity of PC9/GR and HCC827/GR cells. We also showed that FOXM1 overexpression attenuated PC9 and HCC827 cell apoptosis and G1 arrest effects of gefitinib, whereas FOXM1 knockdown promoted PC9/GR and HCC827/GR cell apoptosis and G1 arrest effects of gefitinib. In consistent with our findings, it was also reported that FOXM1 had an important role in the resistance of cancer cells to gefitinib [29–32]. However, the molecular mechanisms underlying FOXM1-mediated gefitinib resistance in lung adenocarcinoma cells remains to be elucidated.

To better understand the molecular mechanism by which FOXM1 contributes to gefitinib resistance in lung adenocarcinoma cells, we searched for the potential targets of FOXM1. MET is known to play an important role in lung cancer development as well as gefitinib resistance [33, 34]. Met activation in tumor cells can occur through several molecular mechanisms, such as overexpression, structural alterations, and HGF-dependent or HGF-independent activation [35]. We observed no significant difference in HGF expression between PC9 and PC9/GR cells, and between HCC827 and HCC827/GR cells. However, it has not been fully understood whether and how MET amplification caused by other mechanisms can affect sensitivity to gefitinib. We further investigated whether FOXM1, an oncogenic transcription factor, regulated MET expression via transcription in lung adenocarcinoma cells. The results of the present study showed that FOXM1 bound directly to the MET promoter regions to promote its transcription.

Our findings also suggest the existence of a novel positive feedback loop, which consists of FOXM1 and MET/AKT signaling in lung adenocarcinoma cells. As previously described, AKT is a downstream target of MET signaling and plays a critical role in gefitinib resistance of lung cancer cells [35, 36]. Previous studies also have revealed that the AKT signaling pathway upregulates the expression of FOXM1 in cancer cells [21, 37–39]. Our study demonstrated that FOXM1 activated the MET/AKT signaling pathway by stimulating the transcription of MET in lung adenocarcinoma cells, and this effect was partially inhibited by PHA-665752. Additionally, MET activated and triggered the AKT signaling pathway, which subsequently stimulated FOXM1 expression in lung adenocarcinoma cells, and this effect was partially inhibited by LY294002. Further statistical analysis indicated that the level of FOXM1 is significantly correlated with that of MET and with the activity of the AKT pathway in human lung adenocarcinoma specimens. Constitutive activation of AKT pathway has been detected at high frequency in diverse human cancer cell lines and tissues, including lung cancer [43]. In this study, our results that FOXM1 is upregulated in gefitinib-resistant lung adenocarcinoma cells and FOXM1 can stimulate MET/AKT pathway in a positive-feedback manner strongly suggest that FOXM1 overexpression accounts for a novel mechanism of consecutive MET/AKT activation, which may be a critical mechanism for gefitinib resistance of lung adenocarcinoma cells.

Taken together, FOXM1 lies both downstream and upstream of MET/AKT signaling pathway, and creates a positive feedback loop to promote gefitinib resistance in lung adenocarcinoma cells. Owing to the great importance of the FOXM1/MET/AKT axis in lung adenocarcinoma, our findings strongly indicate that targeting FOXM1 may be a promising therapeutic strategy for overcoming gefitinib resistance in lung adenocarcinoma.

## MATERIALS AND METHODS

### Clinical specimens

This study was approved by the Ethics Committee of the Fourth Military Medical University. A total of 64 pairs of lung adenocarcinoma specimens and adjacent non-cancerous specimens were collected from patients who had underwent resection at Tangdu Hospital Affiliated with the Fourth Military Medical University, with written informed consent of patients. A part of each tissue was immediately snap-frozen in liquid nitrogen while the other part was fixed in formalin for histological examination. None of the patients underwent chemotherapy or other adjuvant treatments before surgery.

### Cell culture and reagents

The PC9 and HCC827 cell lines, which were derived from patients with pulmonary adenocarcinoma carrying an in-frame deletion in EGFR exon 19 (E746-A750 deletion), show strong sensitivity to gefitinib. The PC9 cells and HCC827 cells were obtained from Sigma-Aldrich Corporation (St. Louis, MO) and American Type Culture Collection (ATCC), respectively. The gefitinib-resistant PC9 cell line (PC9/GR) and HCC827 cell line (HCC827/GR) were established by following the protocol described in a previous study [44]. All cells were cultured in RPMI 1640 with 10% fetal bovine serum (Gibco BRL, Gaithersburg, MD), 100 U/mL penicillin (Invitrogen, Carlsbad, CA), and 100 mg/mL streptomycin, and were incubated at 37°C in 5% CO_2_ humidified air. Gefitinib was obtained from AstraZeneca and dissolved in DMSO at various concentrations and quantities, per the experimental design. The specific PI3K/AKT inhibitor LY294002 was purchased from Cell Signaling Technology and was used at a final concentration of 10 μM. The specific MET inhibitor PHA-665752 was purchased from Selleck Chemicals (Houston, TX) and was used at a final concentration of 0.1 μM.

### Plasmids, shRNA and transfection

The pcDNA3.1-FOXM1 (FOXM1) and pcDNA3.1-MET (MET) plasmids were constructed in our laboratory. The shRNA for FOXM1 (shFOXM1), MET(shMET) and negative control shRNA (shNC) were obtained from Santa Cruz Biotechnology (Dallas, TX). Transfections were performed with Lipofectamine 2000 reagent (Invitrogen, Carlsbad, CA) using 1–2 mg of expression vector/ml serum-free medium as described by the manufacturer.

### Growth inhibition assay

Cell survival was measured by 3-(4, 5-dimethylthiazol-2-yl)-2, 5-diphenyltetrazolium bromide (MTT) assay. Untransfected or transfected cells were re-seeded into 96-well plates and incubated at 37°C in humidified 5% CO_2_ for 48 hrs. Serially diluted gefitinib was added, and the cells were incubated for an additional 72 hrs. The absorbance at 490 nm (A490) of each well was measured on a spectrophotometer. Three independent experiments were performed in quadruplicate.

### EdU proliferation assay

To measure cell proliferation, an EdU (5-ethynyl- 2′-deoxyuridine) proliferation assay was performed as described previously [45]. The cells were plated in 24-well plates at a density of 5 × 10^4^ cells/well, then treated with 0.1 μM gefitinib for 72 hrs. Cells were washed with PBS, then incubated in serum-free DMEM containing 10 μmol/L EdU (RiboBio, Guangzhou, China) for 2 hrs. Cells were fixed, then underwent Apollo staining and DNA staining, according to the manufacturer's instructions to detect the number of cycling cells during the EdU treatment. The cells were imaged using fluorescence microscopy, and the number of proliferating cells was averaged to calculate the labeling index.

### Flow cytometry for apoptosis and cell cycle analysis

The cells transfected with pcDNA3.1, FOXM1, shFOXM1 or shNC were treated with 0.1 μM gefitinib for 72 hrs and harvested. For the cell cycle assay, cells were washed with ice-cold phosphate-buffered saline (PBS) and fixed with ice-cold 70% ethanol overnight at –20°C. Fixed cells were rehydrated in PBS for 20 min and subjected to PI/RNase staining. Flow cytometry was performed using a FACScan instrument (Becton Dickinson, Franklin Lakes, NJ), and analysis was performed with CellQuest software (Becton Dickinson, Franklin Lakes, NJ). For cell apoptosis analysis, apoptotic cells were detected with the Annexin V-FITC Apoptosis Detection Kit (Oncogene Research Products, Boston, MA) according to the manufacturer's instructions and analyzed using flow cytometry (Becton Dickinson, Franklin Lakes, NJ).

### Quantitative real-time PCR

Total RNA isolation from cell lines and tissues was performed using Trizol (Invitrogen, Carlsbad, CA). A reverse transcription reaction was performed using a reverse transcription kit (Applied Biosystems, Foster City, CA). Quantitative real-time PCR (qRT-PCR) was performed on an ABI 7500 real-time system (Applied Biosystems, Foster City, CA) according to the manufacturer's protocol. Data were analyzed according to the comparative Ct method [46]. The β-actin was used as an internal control for each specific gene. Three independent experiments were performed to analyze the relative gene expression. Primer sequences are listed in Supplementary Table S1.

### Western blot analysis

Cells were harvested and homogenized with lysis buffer, and western immunoblotting was performed using standard procedures. Total protein was separated on 10% SDS-PAGE gels and transferred to PVDF membrane (Millipore, Bedford, MA). The membrane was blocked with 5% nonfat dry milk in TBS, then probed with the antibody against FOXM1 (Santa Cruz Biotechnology, Dallas, TX), pAKT (Abcam, Cambridge, MA), AKT (Abcam, Cambridge, MA), MET (Abcam, Cambridge, MA), pMET (Abcam, Cambridge, MA) and tubulin (Abcam, Cambridge, MA). After washing, horseradish peroxidase-conjugated anti-rabbit IgG (Santa Cruz Biotechnology, Dallas, TX) was used as a secondary antibody and incubated for 1 h at room temperature. Quantification of band intensity was performed using Image J software.

### Immunohistochemistry

The immunostaining technique was conducted as described previously [22]. The intensity of staining was scored as 0 (negative), 1 (weak), 2 (medium) or 3 (strong), while the extent of staining was scored as 0 (0% of cells stained), 1 (1–25%), 2 (26–50%), 3 (51–75%) or 4 (76–100%). The scores of each tumor sample were multiplied to give a final score of 0–12.

### Chromatin immunoprecipitation assay

Chromatin immunoprecipitation (ChIP) assays were performed in lung adenocarcinoma cells following the protocol provided by the manufacturer (Millipore, Bedford, MA). Briefly, after cross-linking with formaldehyde at 1% final concentration for 10 min at 37°C and the reaction was quenched by addition of glycine to a final concentration of 0.125 M. The cells were lysed in SDS buffer and the pellet was resuspended in nuclei lysis buffer and sonicated. Immunoprecipitation was carried out with FOXM1 antibody (Santa Cruz Biotechnology, Dallas, TX). The PCR primer sequences for DNA fragments as parts of the targeted promoters are provided in Supplementary Table S2.

### Luciferase reporter assay

The luciferase assays were performed using a luciferase assay kit (Promega, Madison, WI) according to the manufacturer's protocol. Cells were plated in 24 well plates and transiently transfected with pGL3-MET vector and Renillar luciferase reporter with FOXM1 shRNA, pcDNA3.1–FOXM1 or control vector using Lipofectamine 2000 (Invitrogen, Carlsbad, CA). Relative firefly luciferase activity was measured using a dual luciferase assay system (Promega, Madison, WI) 24 hours after transfection.

### Xenograft model

BALB/C nude mice aged 4–6 weeks old were purchased from Shanghai Laboratory Animal Center (SLAC, Shanghai, China) and housed within a dedicated SPF facility at the Laboratory Animal Center of the Fourth Military Medical University. All studies were performed following guidelines approved by the Institutional Animal Ethics Committee of the Fourth Military Medical University. For tumor growth evaluation, PC9/GR cells (5 × 10^6^) were stably transfected with shNC or shFOXM1 and subcutaneously injected into the left flank of nude mice. Xenograft size was measured daily and tumor volume was determined as (length × width^2^)/2. After one week, mice were subjected to oral treatment with saline or gefitinib (20 mg/kg) every day. The mice were humanely killed on day 28, and subcutaneous tumors were surgically excised, weighed and photographed. Immunohistochemistry, western blotting and qRT–PCR were performed for Ki67, FOXM1, pAKT and MET.

### Statistical analysis

All data were expressed as mean ± standard deviation (S.D.), and then processed using GraphPad Prism v5.0 software. Data were analyzed using the Student *t-test*. Pearson correlation coefficient was used to measure the strength of the association between FOXM1, pAKT and MET expression levels. Statistical significance was accepted at *P value* less than 0.05.

## SUPPLEMENTARY MATERIALS TABLES


