# Supplementary material for: FOXM1 confers resistance to gefitinib in lung adenocarcinoma via a MET/AKT-dependent positive feedback loop
Source: Oncotarget. 2016 Aug 3;7(37):59245–59. doi: 10.18632/oncotarget.11043 (PMC5312309; doi:10.18632/oncotarget.11043)
Supplement: Supplementary file 1 [file oncotarget-07-59245-s001.pdf]

FOXM1 confers resistance to gefitinib in lung adenocarcinoma via a MET/AKT-dependent positive feedback loop

Supplementary Materials

Supplementary Table S1: Sequence of primers for quantitative real-time PCR

Gene	Primer sequence
FOXM1	
Forward	5′-CACCCCAGTGCCAACCGCTACTTG-3′
Reverse	5′-AAAGAGGAGCTATCCCCTCCTCAG-3′
MET	
Forward	5′-CCATCCAGTGTCTCCAGAAGTG-3′
Reverse	5′-TTCCCAGTGATAACCAGTGTGTAG-3′
GAPDH	
Forward	5′-GGTGAAGGTCGGAGTCAACG-3′
Reverse	5′-CAAAGTTGTCATGGATGHACC-3′

Supplementary Table S2: Genes and primer sequences for ChIP assay

Gene	Primer sequence
MET	
Forward	5′-AGGAACAGGAAAATCTCG-3′
Reverse	5′-GCATAACTAAAAGTATCTGGG-3′
